# Methods of a New Chronic Pancreatitis and Spontaneous Pancreatic Cancer Mouse Model Using Retrograde Pancreatic Duct Injection of Dibutyltin Dichloride

**DOI:** 10.3389/fonc.2022.947133

**Published:** 2022-07-06

**Authors:** Deyu Zhang, Wanshun Li, Meiqi Wang, Hua Yin, Chuanchao Xia, Keliang Li, Haojie Huang

**Affiliations:** ^1^Department of Gastroenterology, First Affiliated Hospital, Naval Medical University, Shanghai, China; ^2^Academy of Medical Sciences, Zhengzhou University, Zhengzhou, China

**Keywords:** pancreatic cancer, DBTC, pancreatic disease, chronic pancreatitis, mouse model

## Abstract

The current study aimed to develop a new chronic pancreatitis and spontaneous pancreatic cancer model on C57/BL6 mouse through retrograde pancreatic duct injection of dibutyltin dichloride (DBTC) and explore its basic pathological changes as compared to the previous published chronic pancreatitis model through tail vein injection of DBTC with alcohol drinking. C57/BL6 mice were randomly divided into 3 groups: CG (control group; n = 15), VG (tail vein injection of DBTC (8 mg/kg) with 10% alcohol drinking group; n = 20), and PG (retrograde pancreatic duct injection of DBTC group (1 mg/kg); n = 30). Five mice in each group were sacrificed at a specific time point after the first treatment. The pathological section was observed. The activities of amylase, bilirubin, and hyaluronic acid in serum were determined. The expression of fibronectin, COL1A1, α-SMA, MMP-1, and TIMP-1 in the pancreas was assayed. Severe fibrosis of the pancreas with inflammatory cell infiltration could be observed on day 21 in the PG. In the VG, slight fibrosis of the pancreas with inflammatory cell infiltration was observed on day 28. There were significant differences in serum amylase, bilirubin, and hyaluronic acid levels between the PG and VG. The protein level of COL1A1 and α-SMA significantly increased in the PG. The mRNA expression of TIMP-1 is upregulated and the MMP-1 mRNA level is downregulated in the PG. Finally, typical neoplastic pathological change is significantly obvious in the PG. In conclusion, we established and validated a new chronic pancreatitis (CP) and spontaneous pancreatic cancer mouse model through retrograde injection of DBTC into the pancreatic duct. Previously reported mouse model through tail vein injection of DBTC with alcohol drinking could not cause obvious CP and neoplastic pathological change in mice.

## Introduction

Chronic pancreatitis (CP) is a pancreatic disease mainly manifested by upper abdominal pain, diabetes, malnutrition, and diarrhea (1, 2). Additionally, CP is a significant pathogenic factor for pancreatic cancer. The main causes of CP are hereditary susceptibility, biliary tract obstruction, alcohol abuse, and hyperlipidemia (3, 4). The global morbidity of CP has surged to around 5 cases per 10,000 persons in 2019. It is vital for us to identify the underlying pathophysiology and to develop specific new therapeutic methods for CP and CP-induced pancreatic cancer (5).

The major pancreatic pathology of CP is characterized by extensive interstitial fibrosis, continuous inflammatory cell infiltration, and irreversible endocrine function damage. It has been shown that activated pancreatic stellate cells are the main source of pancreatic extracellular matrix proteins and the main effector cells of pancreatic fibrosis (6). Human CP tissue could be difficult to be acquired because surgery cannot be used to treat CP (5). In addition, chronic pancreatic inflammation is an obvious risk factor for the occurrence of pancreatic cancer, and the same fibro-inflammatory reaction is observed in pancreatic cancer, together with a loss of normal pancreatic cells.

Mouse models are commonly employed to study the progression of CP and pancreatic cancer, with genetic and pharmacological tools used to elucidate the cellular and cellular interactions within pancreatic tumors (7). The widely used CP and spontaneous pancreatic cancer mouse model is the intermittent intraperitoneal injection of caerulein. Caerulein, a peptide analog of cholecystokinin (CCK) and gastrin, stimulates pancreatic exocrine secretion and increases pancreatic proteolytic enzyme secretion to levels that cause injury of pancreatic acinar (8). In the 1990s, Van Laethem et al. reported the successful use of a 4-week intermittent intraperitoneal caerulein injection to induce a typical CP model (9). This method is consistent with the pathogenesis of CP and is widely used to investigate the pathogenesis and pathophysiological changes of CP (10). However, caerulein is expensive, so there is still a need for another effective CP and a spontaneous pancreatic cancer mouse model to corroborate the related research.

Tail injection of dibutyltin dichloride (DBTC) could be another feasible way to establish a CP mouse model. In early 1999, Merkord et al. illustrated that tail injection of DBTC with alcohol drinking could successfully induce CP in rats (11). However, the feasibility of the use of tail injection of DBTC in mice remains unclear, and some results seem to be contradictory. On the one hand, Zhang et al. reported the successful establishment of the CP mouse model through tail injection of DBTC with alcohol drinking (12). On the other hand, Lütt et al. illustrated that tail injection of DBTC with alcohol drinking could not induce CP in mice (13). Furthermore, only the caerulein-induced CP mouse model was confirmed to be used as a spontaneous pancreatic cancer mouse model. The viability of other CP models to be used as a spontaneous pancreatic cancer mouse model has not been clarified (14).

In our current study, a viable novel CP and spontaneous pancreatic cancer mouse model through retrograde injection of DBTC in pancreatic duct on mice was established and analyzed. In addition, we also analyzed the feasibility of the contradictory CP mouse model, which is a tail injection of DBTC with alcohol drinking, and we compared the efficacy and neoplastic changes between these two methods.

## Materials and Methods

### Genetically Engineered Mice

LSL-K-Ras^G12D^ male mice, which possess the conditional knock-in mutant K-Ras^G12D^, were obtained from Pro. Craig D. Logsdon (15). Ela-CreERT mice, which express tamoxifen-regulated CreERT specifically in pancreatic acinar cells under the control of a full-length elastase gene promoter, were developed in our laboratory as previously described (16). For targeted expression of K-Ras^G12D^ in pancreatic acinar cells, LSL-K-Ras^G12D^ mice were bred with Ela-CreERT mice to generate LSL-K-Ras/Ela-CreERT double-transgenic mice (acinar-Ras mice). Before experiments, tamoxifen was orally administered to these mice to activate Cre recombination in pancreatic acinar cells. The mice were raised in the Gastroenterology Laboratory of Changhai Hospital with standard food and a specified water supply.

### Chronic Pancreatitis Induction

The mice were randomly divided into 3 groups: CG (control group), VG (tail vein injection group), and PG (retrograde pancreatic duct injection group) (n = 60). DBTC was solved in 60% ethanol, 20% glycerin, and 20% physiological saline. The DBTC concentration in the tail vein was 3.2 μg/μl. The DBTC concentration of retrograde pancreatic duct injection was 0.5 μg/μl. Mice in the VG were fed with 10% alcohol after DBTC injection in the tail vein. Control mice were only injected with solvent (60% ethanol, 20% glycerin, and 20% normal saline) through the retrograde pancreatic duct.

### Preparation of Serum and Tissue Samples

Serum samples were collected from the inner canthus vein and stored at −80°C for the detection of amylase, hyaluronic acid, and bilirubin. The histological morphology of the pancreas was observed by H&E staining, and the degree of pancreatic fibrosis was observed by Masson staining. F4/80 and CD3 were detected by immunohistochemistry, and fibronectin (FN) was detected by immunofluorescence. COL1A1 and α-SMA contents were detected by Western blotting. The expression of matrix metalloproteinase-1 (MMP-1) mRNA and tissue inhibitor of metalloproteinases-1 (TIMP-1) mRNA was detected by real-time PCR.

### Histological Analysis

The pancreas of mice was removed, then fixed with 4% paraformaldehyde, embedded in paraffin, and finally sectioned (5 μm). H&E and Masson staining processes were performed on the sections of the specimens. The morphological changes in the pancreas and the degree of fibrosis were observed by light microscopy.

### Western Blotting

Pancreatic tissues were collected and dissolved in radioimmunoprecipitation assay (RIPA) buffer and then incubated at 4°C. The solution was centrifuged at 14,000*g* for 10 min at 4°C, and the supernatant was collected. The protein concentration was determined by a standard protein assay kit. The samples were boiled at 98°C for 8 min. Equal amounts of protein (20 μg) were added to the chamber, then subjected to sodium dodecyl sulfate (SDS)–polyacrylamide gel electrophoresis, and blotted to polyvinylidene difluoride (PVDF) membranes. The membranes were blocked in 3% bovine serum albumin (BSA) for 30 min at 4°C and then incubated with an antibody against α-SMA (catalog # 56856, Cell Signaling Technology, Danvers, MA, USA) and COL1 (catalog # 66948, Cell Signaling Technology). The size of detected bands was quantified by using ImageJ software.

### Immunohistochemistry

Paraffin-embedded pancreas tissue sections were dewaxed and rehydrated twice in PBS for 15 min. The sections were incubated with 0.3% H_2_O_2_ for 15 min to block endogenous peroxidases, washed twice in PBS, then incubated with 30 g/L of BSA for 10 min to prevent non-specific binding of antibodies, and then incubated with anti-CD3 (catalog # 78588, Cell Signaling Technology) or anti-F4/80 (catalog # 25514, Cell Signaling Technology). Finally, diaminobenzidine (DAB) was added as chromogen followed by hematoxylin. The images were observed by a light microscope, and the positive areas were analyzed by ImageJ software.

### Immunofluorescence Assay

For immunofluorescence, slides of pancreas tissue were incubated with anti-FN (catalog # 72943, Cell Signaling Technology) and then counterstained with DAPI. Finally, the slides were observed by a fluorescence microscope.

### Polymerase Chain Reaction

The RNA from pancreatic tissue was isolated with TRIzol reagent (Invitrogen, Carlsbad, CA, USA). The concentration of the RNA was analyzed by using a NanoDrop 1000 spectrophotometer (Thermo Fisher, Waltham, MA, USA) and then transcribed into cDNA by using a high-capacity cDNA reverse transcription kit (Life Technologies, Carlsbad, CA, USA) and amplified. Primer sequences, number of cycles, and annealing temperature are listed in **Table S1**. The expression levels were transformed into standard tubulin content, calculated by the 2^−ΔΔCt^ method.

## Results

### Surgical Method and Pancreatic Weight Change

**Figure 1:**
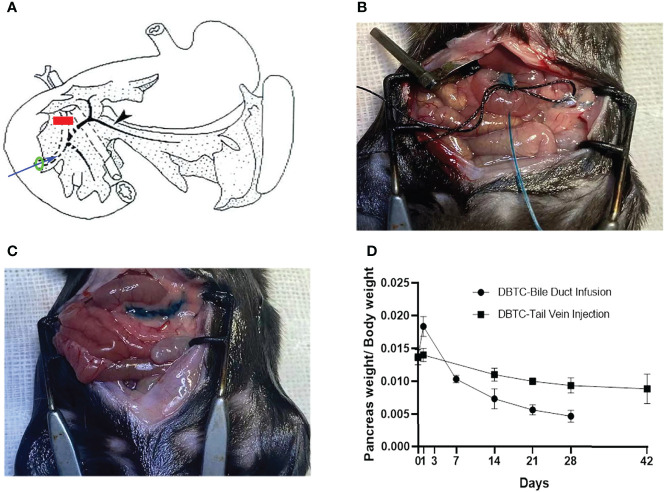
**(A)** Schematic diagram of retrograde pancreatic duct injection of DBTC. Blue arrow, fine catheter to infuse DBTC solution; red line, clip to block distal biliary duct; black arrow, pancreatic duct; green ring, suture to secure biliary duct. **(B)** Intraoperative photographs of retrograde pancreatic duct injection of DBTC (during infusion). **(C)** Intraoperative photographs of retrograde pancreatic duct injection of DBTC (after infusion). Methylene blue (1%) is added to DBTC solution to trace pancreas parenchyma. **(D)** Percentage of pancreas weight/body weight in different groups. DBTC, dibutyltin dichloride.

Surgical methods of retrograde pancreatic duct injection of DBTC (PG) are shown in **Figure 1A**. In brief, after general anesthesia and laparotomy, a fine catheter was inserted into the biliary duct to puncture the intestinal wall. Then, the clip was placed to block the distal biliary duct. A surgical suture was used to secure the proximal biliary duct with a fine catheter, and then infusion was started. Intraoperative photographs of retrograde pancreatic duct injection of DBTC (during infusion and after infusion) are shown in **Figures 1B, C**. The PG shows more significant pancreatic atrophy than the VG in **Figure 1D**.

### Histological Change of Pancreas Between PG and VG

**Figure 2:**
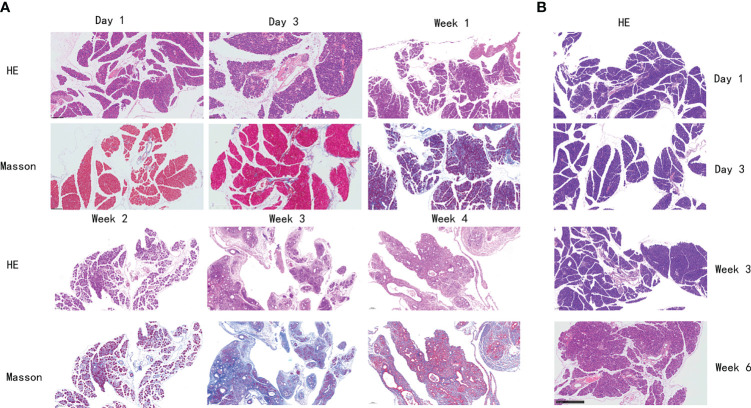
Histological changes of the pancreas in mice with chronic pancreatitis. **(A)** Histological changes of mice in PG pancreas at different time points by H&E and Masson staining. **(B)** Histological changes of mice in VG pancreas at different time points by H&E staining. PG, retrograde pancreatic duct injection group; VG, tail vein injection group.

The histological features of the pancreas of mice in the PG were significantly changed to pancreatic atrophy, inflammatory cell infiltration, and pancreatic tissue fibrosis in 4 weeks (**Figure 2A**). After injection, pancreatic edema immediately appeared in the PG. On day 3, a partial inflammatory cell was observed, dominated by neutrophils and lymphocytes. On day 7, inflammatory cell infiltration became more obvious, and pancreatic cell necrosis also appeared. On day 14, the pancreas parenchyma showed obvious necrosis, with macrophages infiltrating and replacing part of the pancreatic duct epithelial cells. On day 28, pancreatic tissue was extensively replaced by interstitial fibers and connective tissue deposition. However, mice in the VG only showed mild pancreatic edema on day 14 (**Figure 2B**). On day 28, slight inflammatory cell infiltration and local fibrosis were observed. On day 42, although the degree of pancreatic tissue fibrosis was aggravated, it was still far less than that in the PG. Compared with the control group, Masson staining showed a significant increase in collagen deposition in the PG and only a slight increase in the VG, in each respective time point of our study.

### Changes in Serum Amylase, Bilirubin, and Hyaluronic Acid Contents Between PG and VG

**Figure 3:**
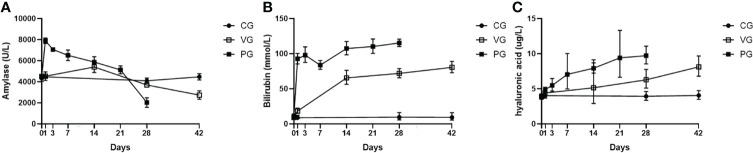
Serum biochemical indices and pancreatic proportion of mice in CG, VG, and PG. **(A)** Serum amylase (U/L). **(B)** Bilirubin level in serum (mmol/L). **(C)** Hyaluronic acid (μg/L). PG, retrograde pancreatic duct injection group; VG, tail vein injection group; CG, control group.

Amylase activity of mice in the PG after injection increased sharply within a day and then began to decline, but still higher than normal, and after 21 days, with extensive fibrosis of the pancreas and massive destruction, amylase activity decreased sharply, and the amylase activity of mice in the VG increased slightly from 1 to 14 days and then decreased after 14 days (**Figure 3A**). The bilirubin levels of mice in the VG and PG increased, indicating that the pathogenetic mechanism of CP was related to liver injury in mice (**Figure 3B**). Serum hyaluronic acid levels and bilirubin levels also increased (**Figure 3C**).

### Change in Inflammatory Cell Infiltration Between PG and VG

**Figure 4:**
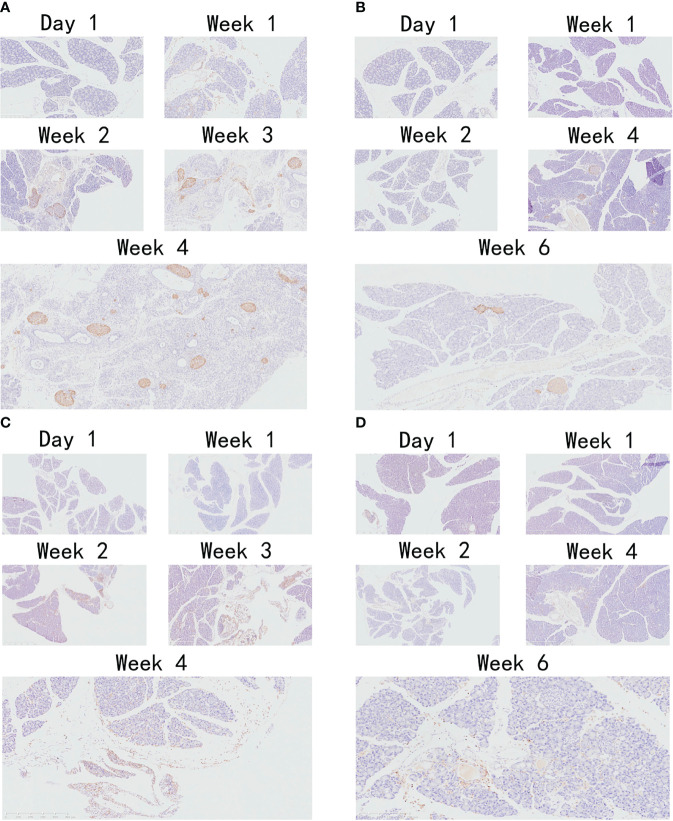
Immunohistochemistry of T lymphocytes. **(A)** T lymphocytes were stained using an anti-mouse CD3 monoclonal antibody in PG. **(B)** T lymphocytes were stained using an anti-mouse CD3 monoclonal antibody in VG. **(C)** Macrophages were stained using an anti-mouse F4/80 monoclonal antibody in PG. **(D)** Macrophages were stained using an anti-mouse F4/80 monoclonal antibody in VG. PG, retrograde pancreatic duct injection group; VG, tail vein injection group.

**Figure 4** shows the results of T lymphocyte and macrophage infiltration. The results suggest that macrophages and T lymphocytes are the main inflammatory cell groups in DBTC-induced CP of the PG. For T lymphocytes, at day 1, T lymphocyte marker CD3 was not found in either the VG or PG. However, after 1 week, a slight T lymphocyte marker CD3 activity was observed in the PG (**Figure 4A**), indicating the presence of T lymphocyte infiltration, but not in the VG. The number of T lymphocytes in the PG increased and remained elevated from day 21 to 28. However, few T lymphocytes were observed in the VG on day 14 (**Figure 4B**), and the number of infiltrated T lymphocytes remained much lower than in the PG throughout the observation period. For macrophages, mild macrophage infiltration was observed in the VG (**Figure 4C**) and PG (**Figure 4D**) on day 14, but as time went on, the number of macrophages in the PG increased faster than that in the VG, and it was obvious that the number of macrophages in the PG at day 28 was much more than that in the VG at day 42.

### Fibrotic Parameter Changes in Pancreas

**Figure 5:**
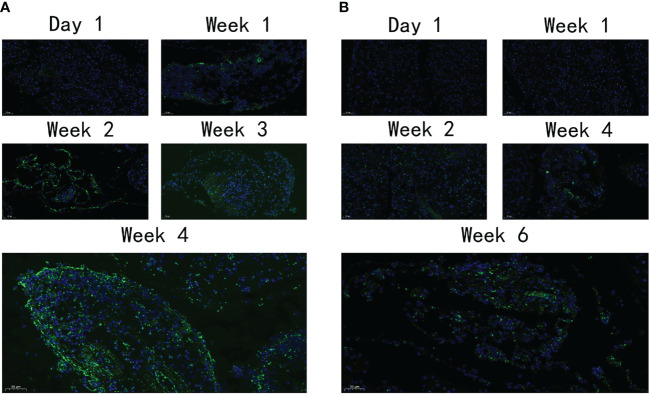
The expression level of FN. **(A)** The expression of FN in PG at different time points. **(B)** The expression of FN in VG at different time points. FN, fibronectin; PG, retrograde pancreatic duct injection group; VG, tail vein injection group.

Immunofluorescence was used to detect the distribution and expression of FN, a pivotal fibrosis index. On day 1, FN expression was observed neither in the VG nor in the PG. After 1 week, a moderate amount of FN expression was detected in the PG (**Figure 5A**) and increased throughout the observation period. However, only a moderate amount of FN was observed on day 42 in the VG (**Figure 5B**), indicating that the fibrosis level that appeared in the VG was so low that the mouse model cannot be established.

### Changes in Fibrosis Indicator and Pancreatic Neoplastic Phenotype

**Figure 6:**
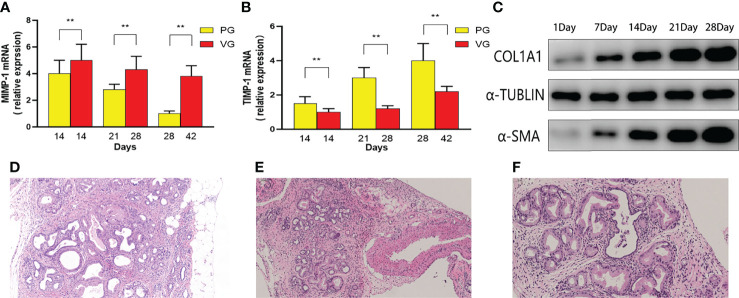
The MMP-1 mRNA and TIMP-1 mRNA contents in mice of PG and VG changed at different time points. **(A)** Changes in MMP-1 mRNA expression (PG vs. VG). **(B)** Changes in TIMP-1 mRNA expression (PG vs. VG). **(C)** The expression levels of α-SMA and COL1 at different time points (PG). **(D–F)** Representative H&E images of PG 1 month after injection. PG, retrograde pancreatic duct injection group; VG, tail vein injection group.

An important feature of severe CP is the disorganized expression of metalloproteinase, including upregulated MMP-1 and downregulated TIMP-1. In our study, MMP-1 and TIMP-1 mRNAs were detected by real-time PCR (**Figures 6A, B**). The decreased rate of MMP-1 and TIMP-1 expression in the PG is sharper than that in the VG, suggesting that CP in the PG is much more severe than that in the VG. After the aforementioned multi-angle analysis, tail vein injection of DBTC with alcohol drinking (VG) has been proven to be unable to induce moderate CP. We further tested the protein level of other fibrotic factors, including COL1A1 and α-SMA, in the retrograde pancreatic duct injection group (PG). COL1A1 and α-SMA expression increased throughout the observation period and surged during the first 21 days (**Figure 6C**). Moreover, a pancreatic neoplastic pathological change could also be identified in 28 days in the PG (**Figures 6D–F**).

## Discussion

CP is a refractory pancreatic disease, mainly caused by biliary dysfunction or alcohol abuse. The pathological manifestation of CP is chronic inflammation in pancreatic parenchyma, atrophy of pancreatic acinar, and fibrous hyperplasia (17, 18). The incidence rate of CP shows a moderate escalation in the past 10 years due to larger alcohol consumption and increased rates of obesity worldwide (5). CP can eventually progress to pancreatic cancer. Therefore, the basic research of CP has attracted great attention from gastroenterologists and scientists. Several effective and reliable rodent models are needed to promote these studies. Additionally, the mouse model could be more flexible and useful than the rat model, because mice are easier to be raised and investigated, and mice have gallbladder in contrast to rats. This can improve the confidence and translational meaning of CP and pancreatic cancer basic research and medical treatment.

DBTC is a fat-soluble substance, and a moderate concentration of DBTC can cause necrosis of pancreatic duct epithelial cells and eventually block the pancreatic duct, resulting in pancreatitis *in vivo*. The blocked pancreatic duct can induce activated pancreatic stellate cells to produce cytokines, resulting in pancreatic fibrosis, and then CP symptoms emerge. This procedure could preferably mimic CP caused by biliary dysfunction in humans, and tail injection of DBTC with alcohol intake has been used in the rat CP model (19). A number of studies based on this rat CP model have been reported (20–22), suggesting the superior robustness of this model. However, DBTC injection has not been widely used in the mouse CP model. The highly toxic DBTC could be an important reason, and the dose used in the rat model could be lethal to mice, as one report has mentioned (23). One interesting question is whether it can successfully induce experimental CP through low-dose injection of DBTC in mice. In the Zhang et al. research, they reported that a low-dose injection of DBTC can induce a classical CP phenotype, including obvious pancreatic acinar atrophy and activation of pancreatic stellate cells and a large ratio of fibrous hyperplasia (12). However, in another research developed by Lütt et al., the researchers reported that the same dose of DBTC through tail injection could not induce an obvious CP phenotype in mice (13). Our finding strongly supports the prospective of Lütt et al.; 6 weeks after tail injection of DBTC with alcohol drinking, we were not able to observe a distinct pancreatic acinar atrophy and interstitial fibrous hyperplasia phenotype, and hyaluronic acid, bilirubin, and amylase contents did not significantly change. The injection dose of our research is consistent with that of a previous study (13). According to some previous studies, an increase in the tail injection dose of DBTC in mice (6–8 mg/kg) could trigger systemic toxicity (13, 23, 24). We can conclude that increasing the dose of DBTC to attempt to induce a CP phenotype can be unacceptable and that tail injection of DBTC with alcohol intake cannot induce an obvious CP-like pathological feature in mice. This finding would end the debate whether tail injection of DBTC with alcohol could induce CP and could be an ideal CP model in mouse.

Another highlight is a viable CP mouse model through retrograde pancreatic duct injection of DBTC has been established and validated in our current research. This mouse model showed great robustness for formatting classical CP pathological features in less than 4 weeks and a 100% survival rate. After 4 weeks, fibrosis in pancreas parenchyma and pancreatic acinar atrophy can be clearly observed in PG mice. The protein level of fibrosis biomarkers in the pancreas, including FN, COL1A1, and α-SMA, were found to be significantly increased. In addition, serum hyaluronic acid, bilirubin, and amylase contents show an ascending tendency in the initial 14 days. Then, with the progress of damage in pancreatic tissue with further loss of endocrine and exocrine functions, amylase levels began to decline while bilirubin and hyaluronic acid levels continued to rise after 14 days. Morphological changes in the pancreas also confirmed that retrograde injection of DBTC through the pancreatic duct can cause classic changes of CP, from pancreatic edema and pancreatic acinar cell necrosis in the initial term to persistent inflammation with pancreatic parenchymal interstitial fibrosis in the final term. The DBTC dose in the retrograde injection group (1 mg/kg) is much lower than that in the tail injection group (8 mg/kg). However, the pancreatic CP-like phenotype is much more significant, which may be attributed to the direct effect of DBTC retrograde injection on the pancreas.

CP is an obvious risk factor for pancreatic cancer (25). Nowadays, the major mouse model to study pancreatitis-induced spontaneous pancreatic cancer is a continuous injection of caerulein. Caerulein is a gastric regulatory molecule similar to CCK. It can stimulate excessive secretion of pancreatic acinar cells, impeding the separation of trypsinogen and lysosomal hydrolase in cells, and can be activated in a cathepsin B-dependent manner. Trypsin activity in turn leads to a series of cell damage. The inflammatory response caused by self-digestion of pancreatic tissue recruits inflammatory cells and releases inflammatory factors, which further cause severe local and even systemic inflammation. Continuous stimulation of the pancreas with caerulein would cause chronic inflammation with fibrosis and carcinogenesis, leading to spontaneous pancreatic cancer (26). This mouse model emphasized the role of excessive secretion of trypsin and cellular oxidative stress in the occurrence of pancreatic cancer. However, one important factor of CP is biliary obstruction, and a model of spontaneous pancreatic cancer is needed to mimic this pathology (4). A previous study has demonstrated that DBTC infusion could lead to acute pancreatitis through the obstruction of the biliary duct (11). In our current study, we proved that retrograde injection of DBTC through the pancreatic duct could induce reproducible spontaneous pancreatic neoplasm in mice with Kas mutation, which can be used as a spontaneous neoplasm mouse model in pancreatic cancer research as a supplementary method for caerulein-induced pancreatic cancer.

In conclusion, in this current study, we established and validated a new CP and spontaneous pancreatic neoplasm mouse model through retrograde injection of DBTC in the pancreatic duct. Moreover, we confirmed that tail injection of DBTC could not induce considerable CP or a neoplastic phenotype in mice.

## Data Availability Statement

The original contributions presented in the study are included in the article/**Supplementary Material**. Further inquiries can be directed to the corresponding author.

## Ethics Statement

The animal study was reviewed and approved by The ethics committee of first Affiliated Hospital of Naval Medical University. Written informed consent was obtained from the owners for the participation of their animals in this study.

## Author Contributions

HH designed the project. DZ, WL, and MW exerted the experiment. MW, WL, and HY contributed to the main part of data analysis and prepared the main manuscript. DZ, MW, and KL revised the article and made suggestions to improve the article. DZ, WL, and MW have contributed equally to this work. Final approval of manuscript: All authors.

## Conflict of Interest

The authors declare that the research was conducted in the absence of any commercial or financial relationships that could be construed as a potential conflict of interest.

## Publisher’s Note

All claims expressed in this article are solely those of the authors and do not necessarily represent those of their affiliated organizations, or those of the publisher, the editors and the reviewers. Any product that may be evaluated in this article, or claim that may be made by its manufacturer, is not guaranteed or endorsed by the publisher.
