# Supplementary material for: Methods of a New Chronic Pancreatitis and Spontaneous Pancreatic Cancer Mouse Model Using Retrograde Pancreatic Duct Injection of Dibutyltin Dichloride
Source: Front Oncol. 2022 Jul 6;12:947133. doi: 10.3389/fonc.2022.947133 (PMC9299365; doi:10.3389/fonc.2022.947133)
Supplement: Supplementary file 1 [file Presentation_1.pptx]

## Slide 1
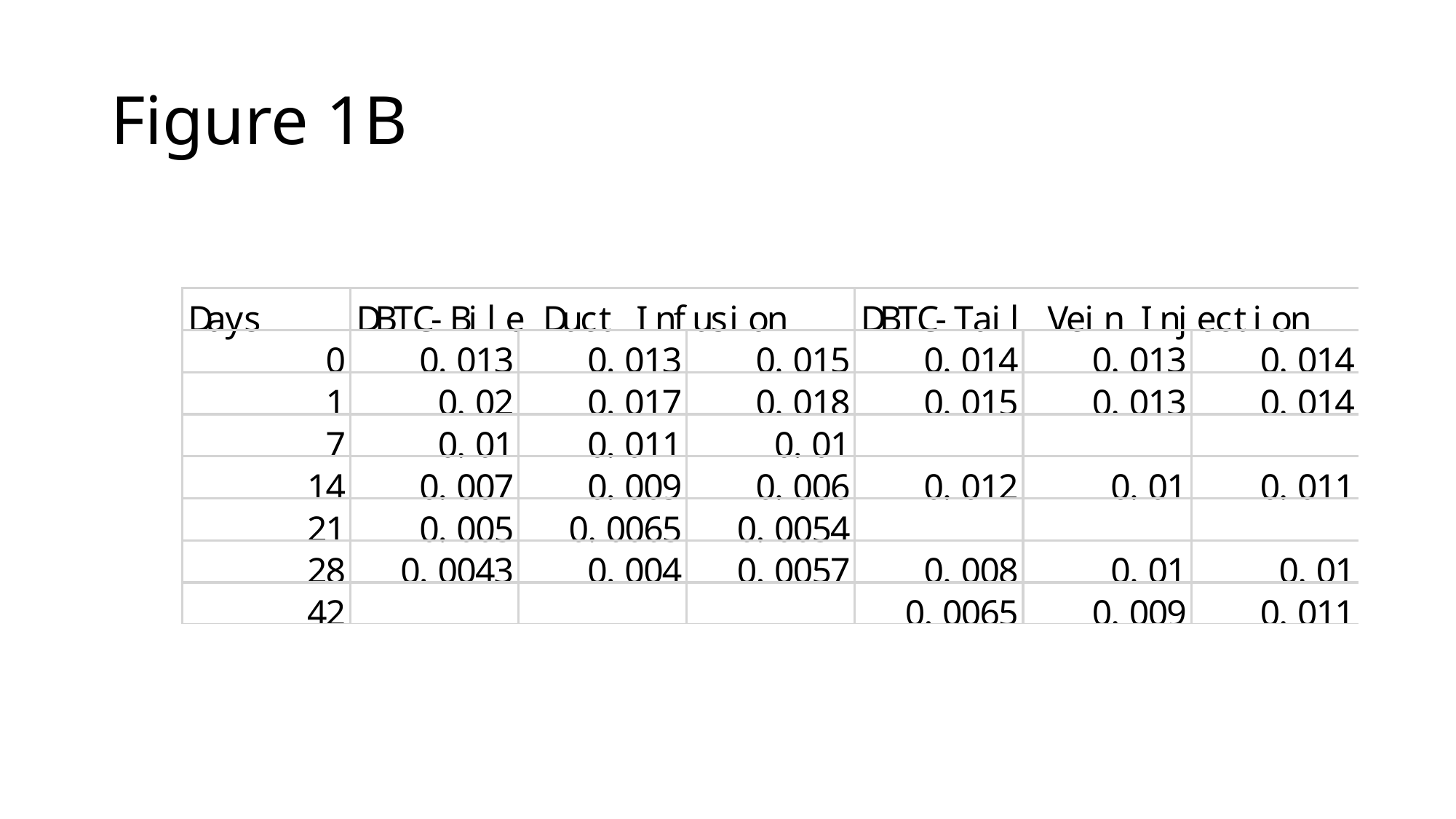

# Figure 1B

## Slide 2
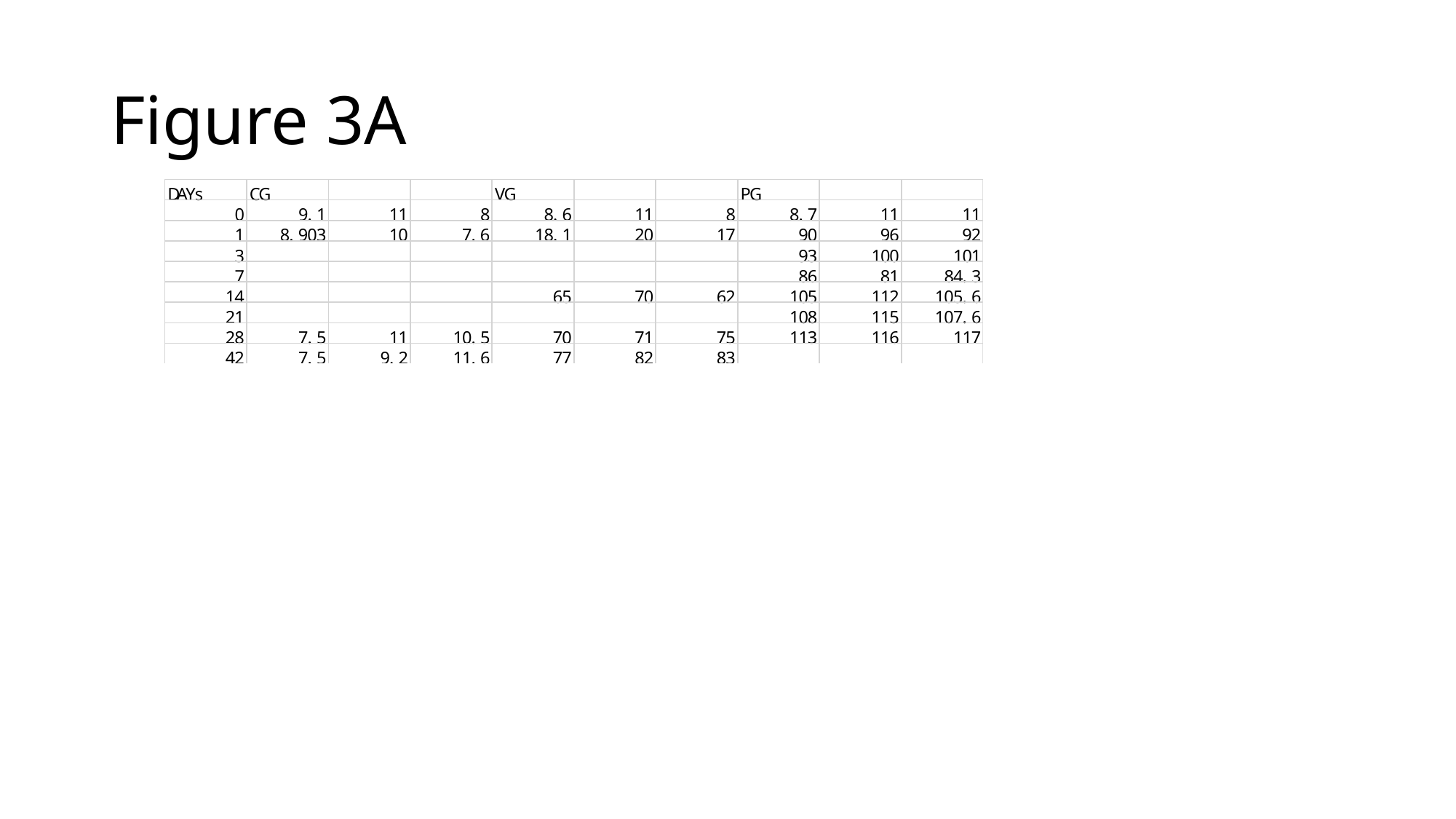

# Figure 3A

## Slide 3
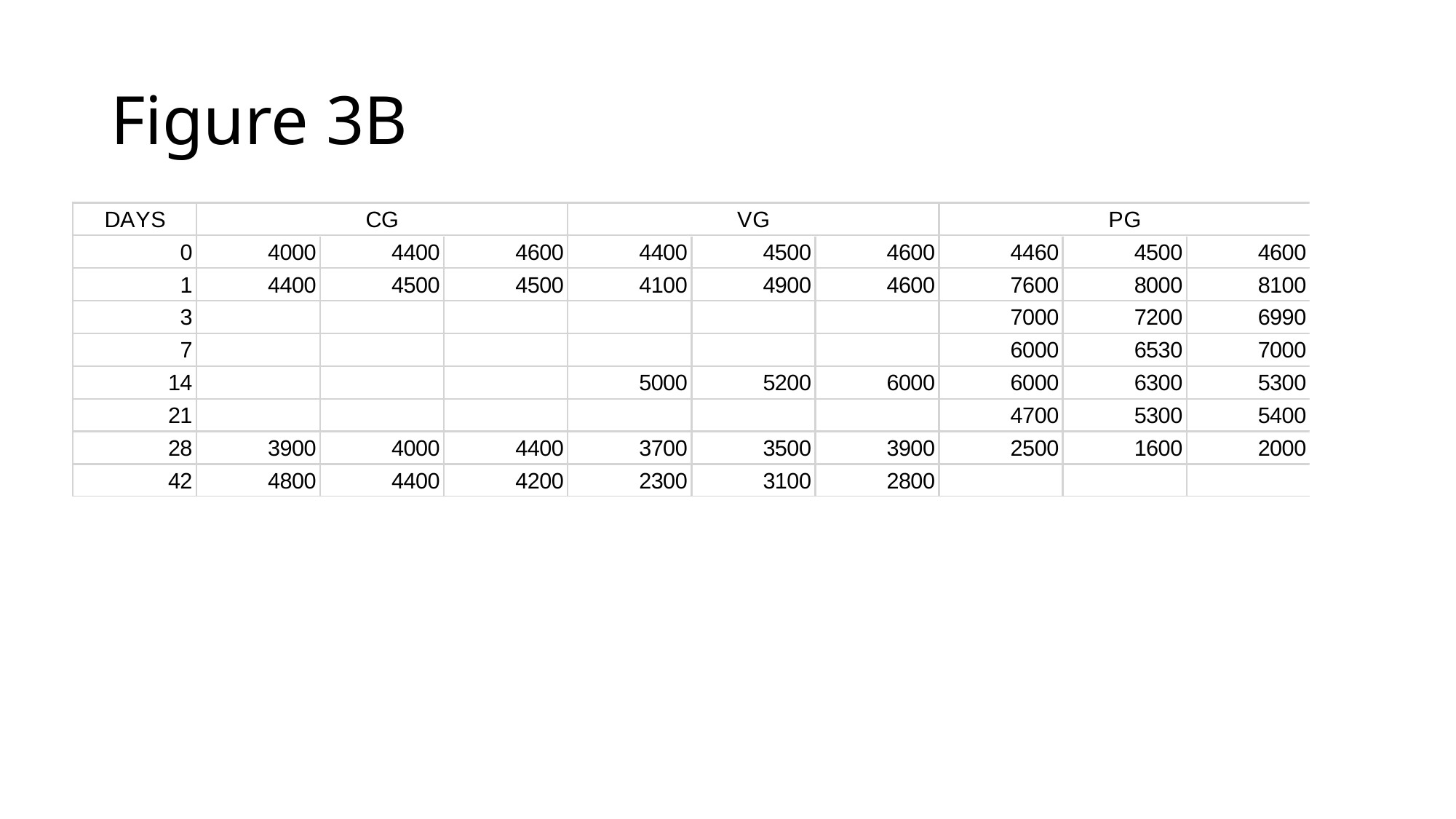

# Figure 3B

## Slide 4
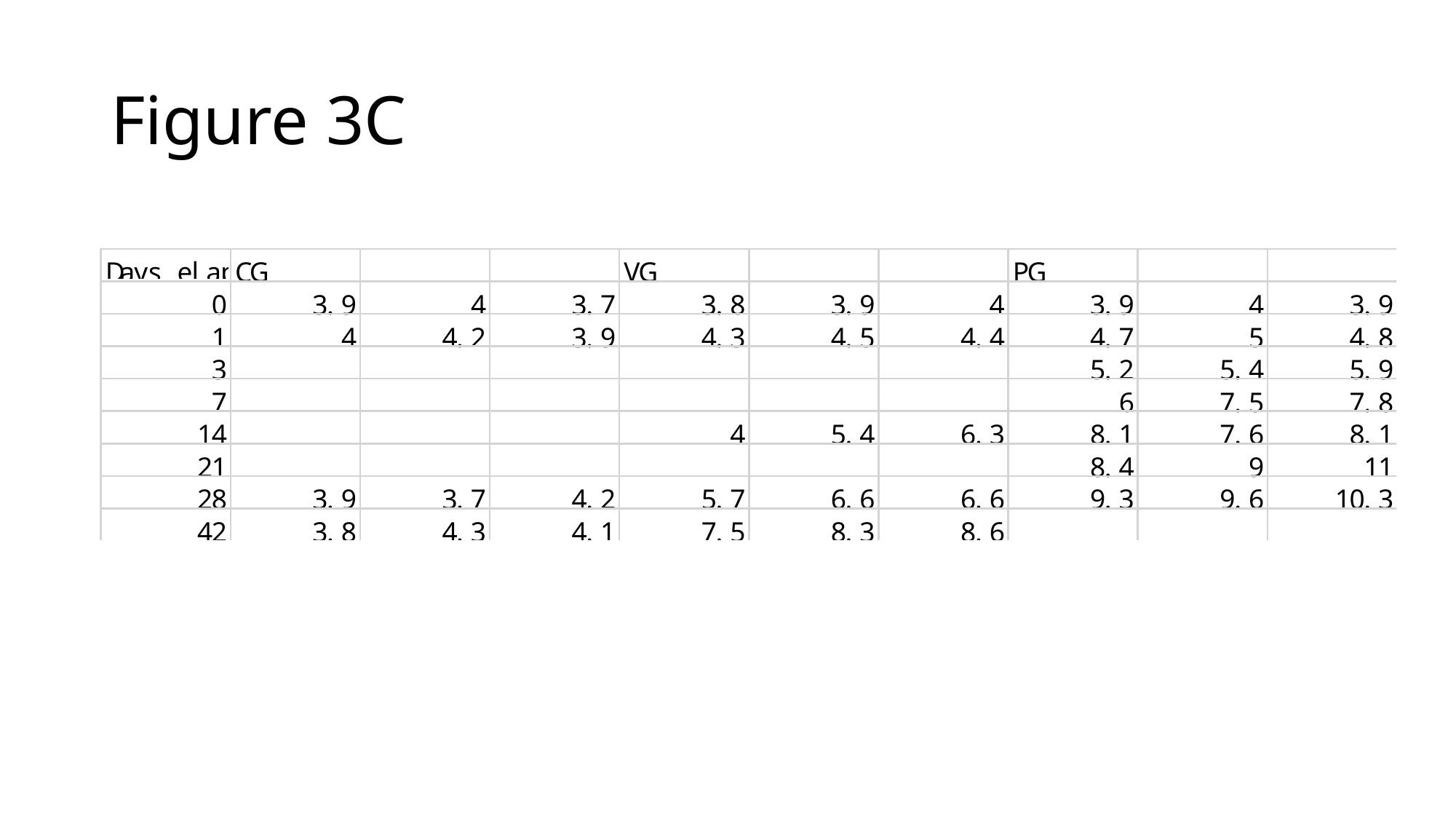

# Figure 3C

## Slide 5
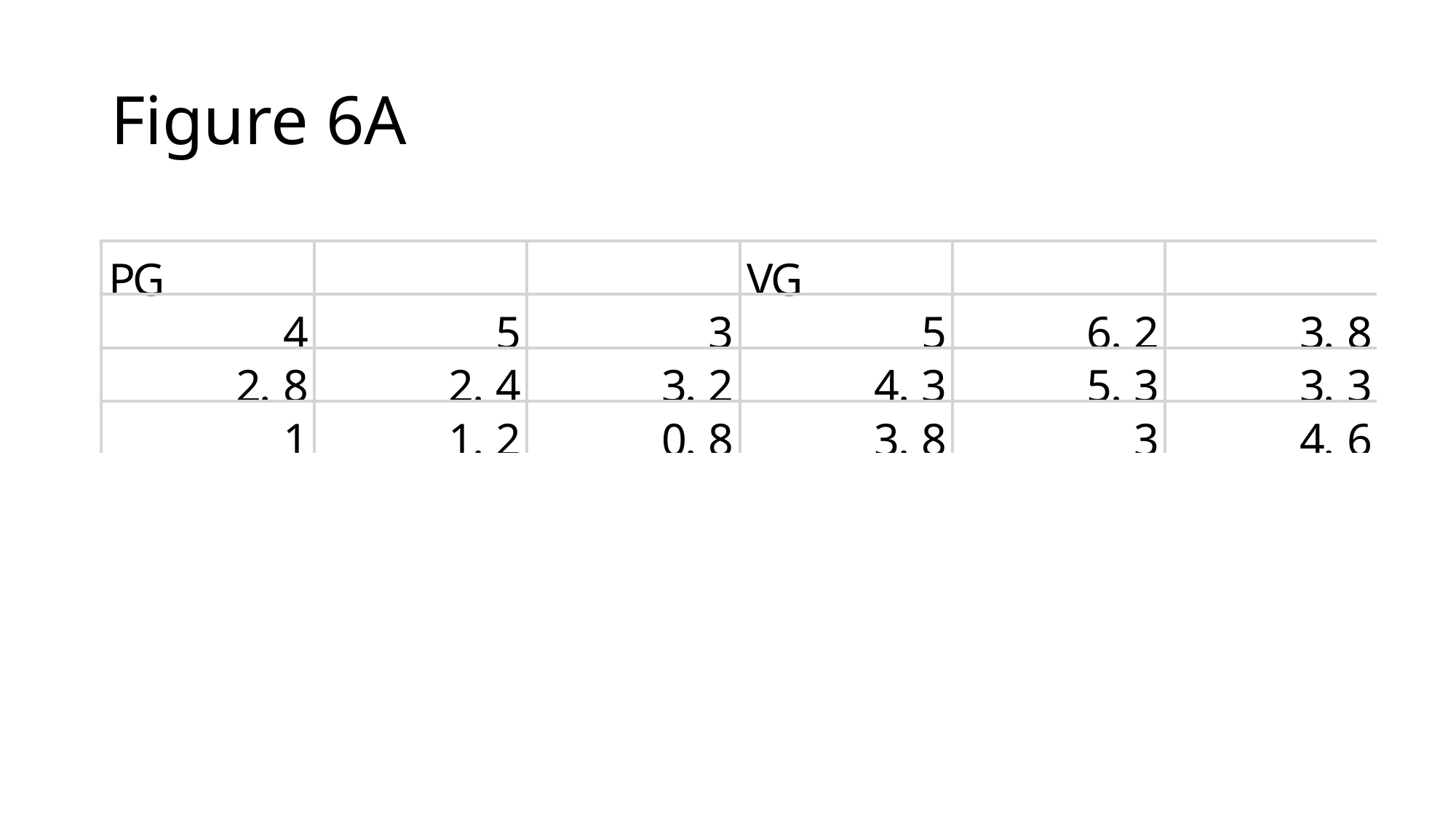

# Figure 6A

## Slide 6
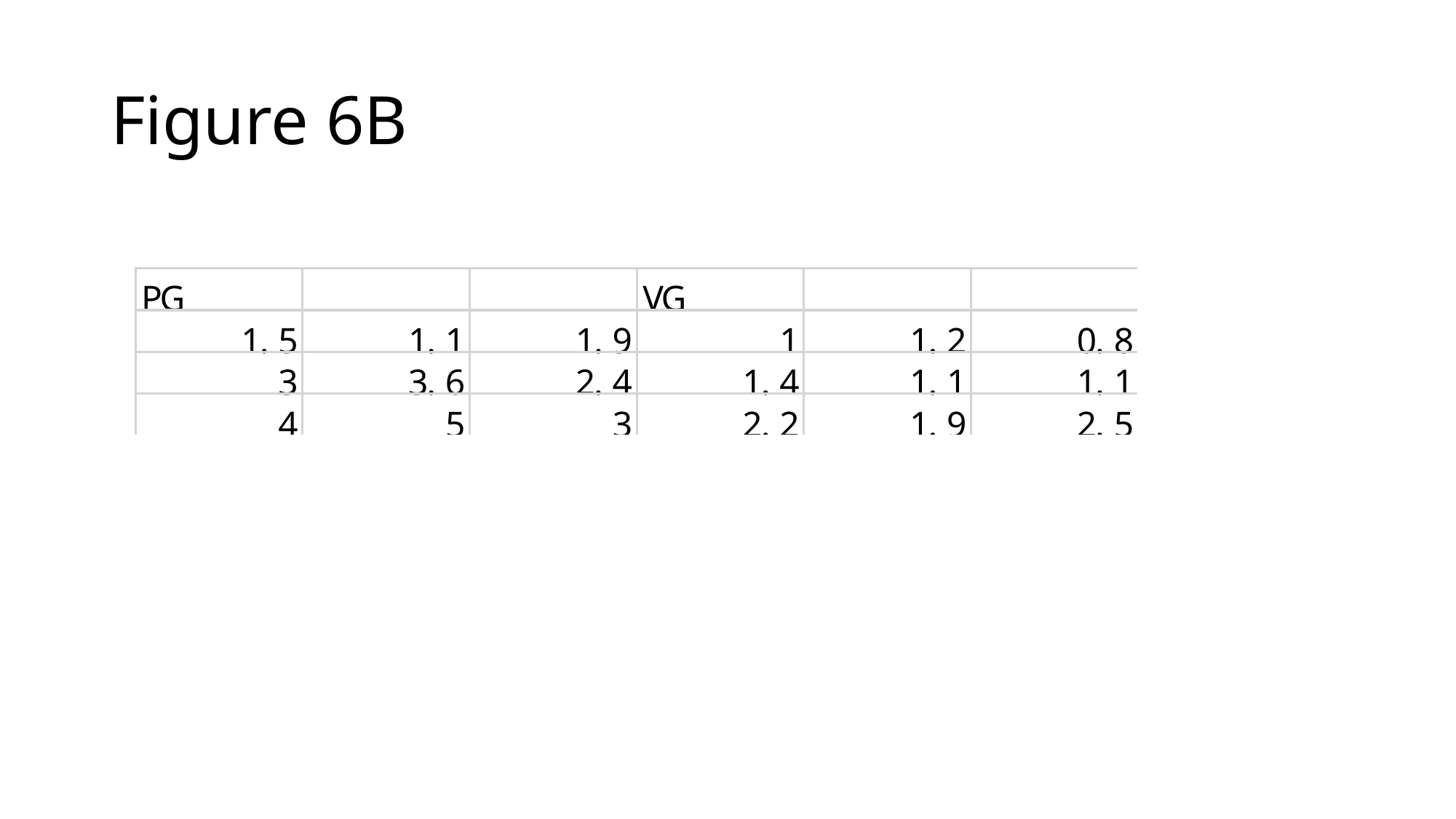

# Figure 6B

## Slide 7
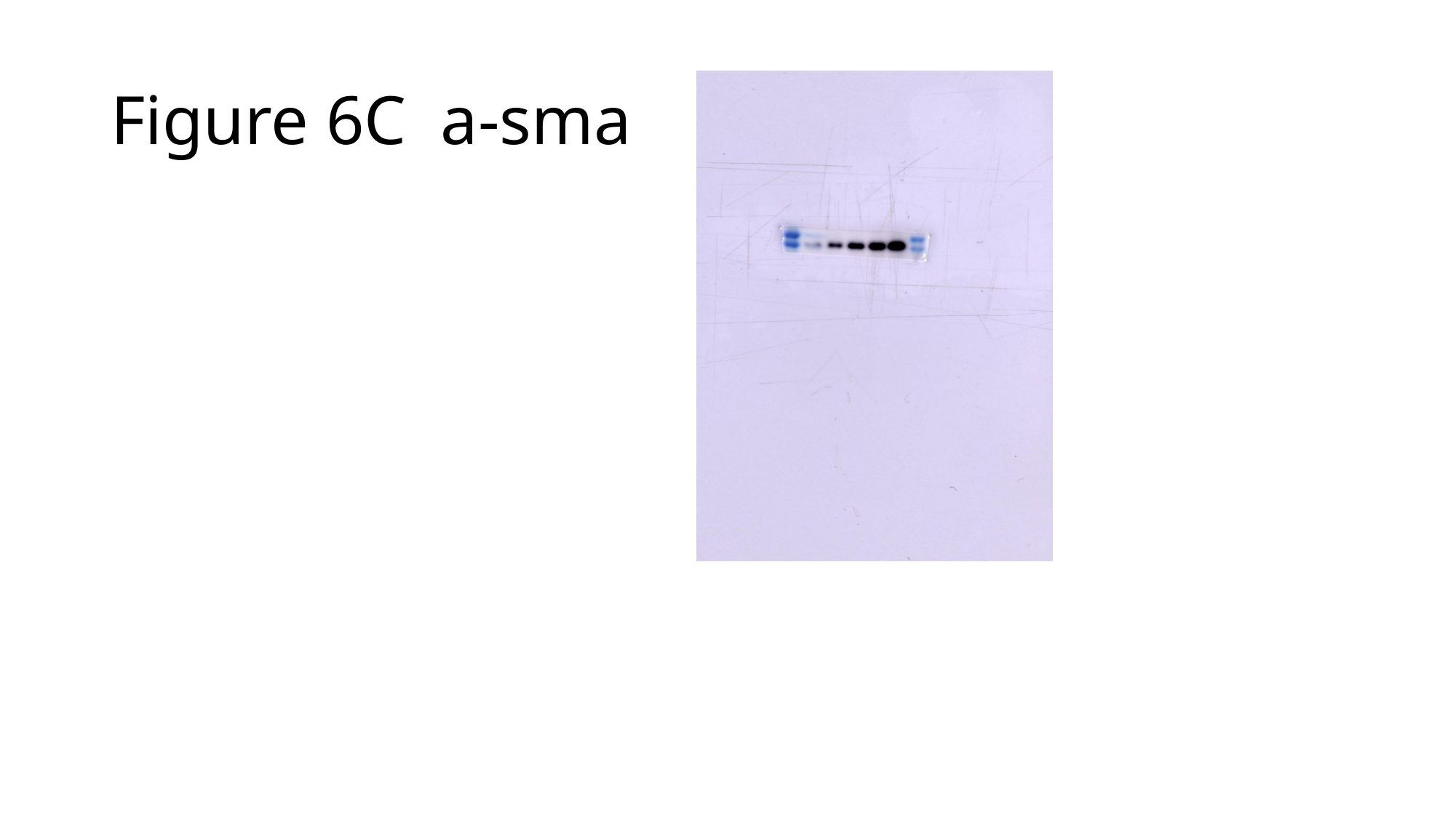

# Figure 6C a-sma

## Slide 8
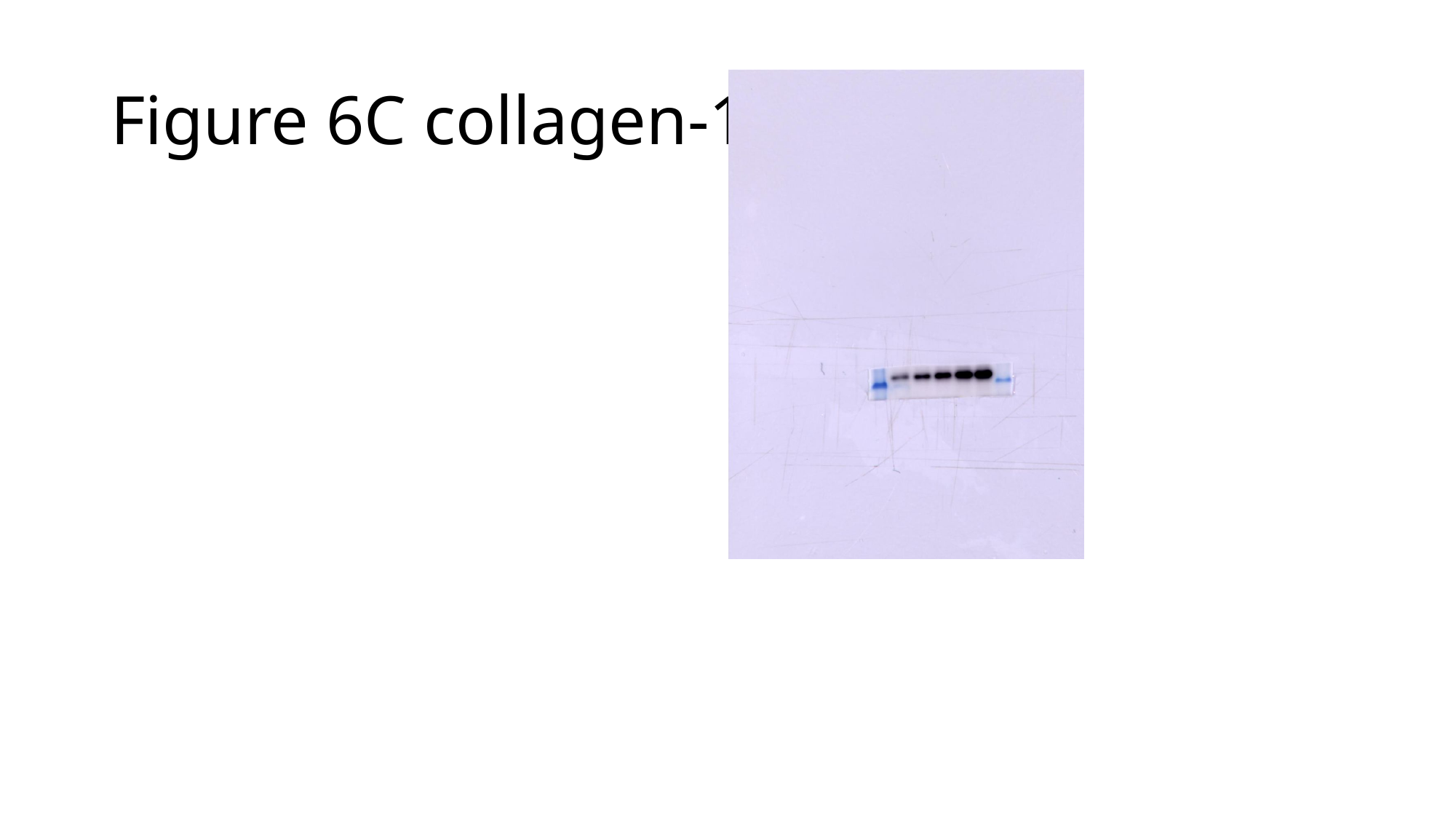

# Figure 6C collagen-1

## Slide 9
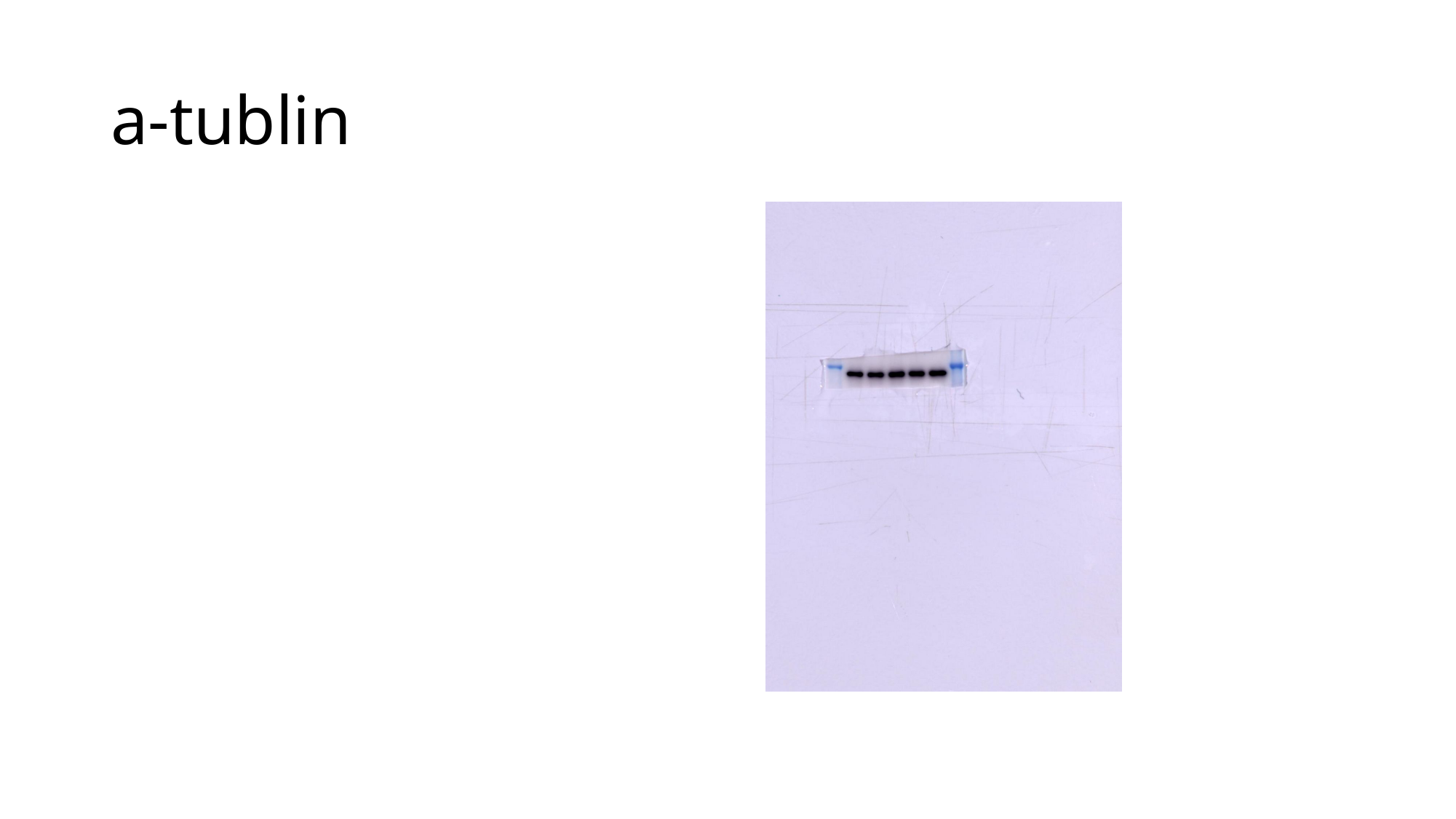

# a-tublin
