# Supplementary material for: Methods of a New Chronic Pancreatitis and Spontaneous Pancreatic Cancer Mouse Model Using Retrograde Pancreatic Duct Injection of Dibutyltin Dichloride
Source: Front Oncol. 2022 Jul 6;12:947133. doi: 10.3389/fonc.2022.947133 (PMC9299365; doi:10.3389/fonc.2022.947133)
Supplement: Supplementary file 2 [file Table_1.docx]

Table S1

Primer sequences used for quantitative polymerase chain reaction

| Gene | Cycles | Tm (℃) | Forward primer | Reverse primer |
| --- | --- | --- | --- | --- |
| S100A6 | 40 | 60 | 5- GCCTCCCTACCGCTCCAA-3 | 5- CACCTCCTGGTCCTTGTTCC-3 |
| S100A9 | 40 | 60 | 5-CAT CTC TGG CAT CTG GCA TCC-3 | 5-CGC TGG TAT AAG GTG GTC TCG-3 |
| S100A12 | 40 | 60 |  |  |
| β-actin | 40 | 60 | 5-ACC ACA CCT TCT ACA ATG AG-3 | 5-ACG ACC AGA GGC ATA CAG-3 |
